# A Fully Automated Post-Surgical Brain Tumor Segmentation Model for Radiation Treatment Planning and Longitudinal Tracking

**DOI:** 10.3390/cancers15153956

**Published:** 2023-08-03

**Authors:** Karthik K. Ramesh, Karen M. Xu, Anuradha G. Trivedi, Vicki Huang, Vahid Khalilzad Sharghi, Lawrence R. Kleinberg, Eric A. Mellon, Hui-Kuo G. Shu, Hyunsuk Shim, Brent D. Weinberg

**Affiliations:** 1 Department of Radiation Oncology, Emory University School of Medicine, Atlanta, GA 30322, USA; karthik.ramesh@emory.edu (K.K.R.); anuradha.trivedi@emory.edu (A.G.T.); vicki.huang@emory.edu (V.H.);; 2 Department of Biomedical Engineering, Emory University and Georgia Institute of Technology, Atlanta, GA 30332, USA; 3 Siemens Healthineers, Atlanta, GA 30329, USA; 4 Department of Radiation Oncology, Johns Hopkins University, Baltimore, MD 21218, USA; 5 Department of Radiation Oncology, Sylvester Comprehensive Cancer Center, Miller School of Medicine, University of Miami, Miami, FL 45056, USA; 6 Winship Cancer Institute, Emory University School of Medicine, Atlanta, GA 30322, USA; 7 Department of Radiology and Imaging Sciences, Emory University School of Medicine, Atlanta, GA 30322, USA

**Keywords:** segmentation, glioblastoma, radiation treatment, longitudinal tracking, deep learning

## Abstract

**Simple Summary:**

With several previous efforts to segment pre-surgical brain tumor lesions from MRI, we sought to shine a different light on the problem. Radiation treatment planning post-surgery still relies heavily on manual contouring of T1-weighted contrast-enhanced and T2-weighted fluid-attenuated inversion recovery MRI. This is one of the first attempts to segment post-surgical brain lesions through deep learning approaches for radiation treatment planning and longitudinal tracking. Our best-performing model segments an overwhelming majority of lesions with at least 70% accuracy and has already been integrated into a web application heavily used by physicians and researchers for longitudinal tracking.

**Abstract:**

Glioblastoma (GBM) has a poor survival rate even with aggressive surgery, concomitant radiation therapy (RT), and adjuvant chemotherapy. Standard-of-care RT involves irradiating a lower dose to the hyperintense lesion in T2-weighted fluid-attenuated inversion recovery MRI (T2w/FLAIR) and a higher dose to the enhancing tumor on contrast-enhanced, T1-weighted MRI (CE-T1w). While there have been several attempts to segment pre-surgical brain tumors, there have been minimal efforts to segment post-surgical tumors, which are complicated by a resection cavity and postoperative blood products, and tools are needed to assist physicians in generating treatment contours and assessing treated patients on follow up. This report is one of the first to train and test multiple deep learning models for the purpose of post-surgical brain tumor segmentation for RT planning and longitudinal tracking. Post-surgical FLAIR and CE-T1w MRIs, as well as their corresponding RT targets (GTV1 and GTV2, respectively) from 225 GBM patients treated with standard RT were trained on multiple deep learning models including: Unet, ResUnet, Swin-Unet, 3D Unet, and Swin-UNETR. These models were tested on an independent dataset of 30 GBM patients with the Dice metric used to evaluate segmentation accuracy. Finally, the best-performing segmentation model was integrated into our longitudinal tracking web application to assign automated structured reporting scores using change in percent cutoffs of lesion volume. The 3D Unet was our best-performing model with mean Dice scores of 0.72 for GTV1 and 0.73 for GTV2 with a standard deviation of 0.17 for both in the test dataset. We have successfully developed a lightweight post-surgical segmentation model for RT planning and longitudinal tracking.

## 1. Introduction

Due to its heavy reliance on imaging, treatment and follow up of brain tumors is an area that could benefit heavily from artificial intelligence (AI) physician guidance. For example, glioblastoma (GBM) is a severe form of brain cancer with a median survival of 15–16 months [1,2,3,4,5]. Standard treatment of GBM first requires resection of as much contrast-enhancing tumor on contrast-enhanced T1-weighted (CE-T1w) MRI as possible. The remaining tumor is treated with adjuvant temozolomide chemotherapy coupled with radiation therapy that targets a high dose of radiation to any residual enhancing tumor in CE-T1w and a lower dose towards hyperintense edema on T2-weighted fluid-attenuated inversion recovery (T2w/FLAIR) MRI. Each of these steps is highly dependent on imaging interpretation, and clinically assistive AI tools could help physicians track and treat GBM patients more accurately and more quickly.

### 1.1. Related Work

**Figure 1:**
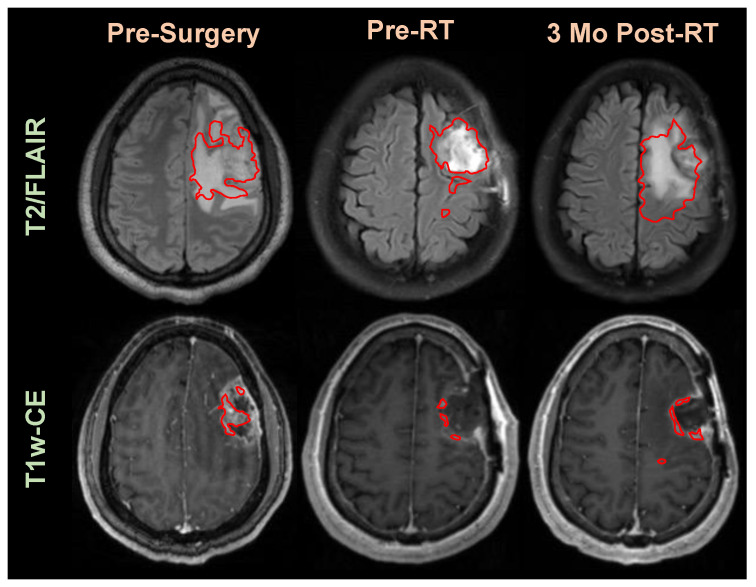
FLAIR and CE-T1w segmentation overlays (in red) from a BraTS competition-winning segmentation model over MRIs prior to surgery, prior to receiving radiation therapy, and 3 months after completing radiation therapy.

While there have been several efforts to segment brain lesions from MRIs, such as through the Brain Tumor Segmentation Challenge (BraTS), those efforts have focused on pre-surgical brain MRIs, and have yet to make a substantial translation into clinically useful tools [6,7,8,9]. There have been minimal efforts to develop segmentation algorithms to assist RT planning after surgery. Post-surgical MRIs have altered morphology that includes a cavity with blood product and heterogenous hyperintensity on FLAIR. In Figure 1, we show an example patient with CE-T1w and T2w/FLAIR MRIs prior to surgery, shortly after completing RT, and three months after completing RT. The CE-T1w MRIs often only contain trace amounts of residual enhancing tumor post-surgery. The cavity coupled with the small remaining lesion leads to a more difficult task for segmentation models to capture the lesion and the entire cavity. Overlaid on each MRI is the segmentation from the highest-performing Swin-UNETR model trained on the BraTS dataset [10]. We only show these segmentations not to critique the model or approach, but to suggest that previous efforts of brain tumor segmentation solve a different problem and may not be generally applicable to treated tumors.

In standard clinical practice, radiation oncologists generate gross tumor volume (GTV) targets that encompass the residual enhancing lesion and cavity in CE-T1w MRI (GTV2) and edema and cavity on T2w/FLAIR (GTV1). Due to the infiltrative nature of GBM, these targets usually have a margin added to generate clinical target volumes (CTVs) and planning treatment volumes (PTVs). There have been publications that focused on cavity segmentation and performing CTV segmentations with GTVs as input [11,12,13]. However, this is one of the first efforts to directly train a model for GTV segmentation. Furthermore, previous brain tumor segmentation approaches utilized four sets of images (T1w-pre contrast, CE-T1w, T2w, and T2w/FLAIR) for segmenting lesions in CE-T1w and T2w/FLAIR. In an effort to develop a lighter-weight model, we are attempting to use only CE-T1w and T2w/FLAIR to perform GTV segmentations. Such models that require fewer inputs could be more clinically translatable and require less effort due to lesser data requirements for segmentation by physicians and researchers.

### 1.2. Purpose

Due to the unique visual morphology of post-surgical lesions, and a clinically unmet segmentation approach for these brain MRIs, we sought to develop a lightweight deep learning algorithm that could assist radiation oncologists in generating RT volumes. Finally, with a sophisticated deep learning segmentation algorithm for post-operative brain tumors, we hope to integrate our algorithm within our web application, the Longitudinal Imaging Tracker (BrICS-LIT), to track post-treatment brain tumors and assign automated structured reporting scores [14]. In Table 1, we summarize the added value of our proposed effort compared to previous approaches of brain lesion segmentation. With BrICS-LIT, physicians can visualize changes in brain lesions over time, as well as assign structured reporting scores such as the Response Assessment in Neuro-Oncology (RANO) and Brain Tumor Reporting and Data System (BT-RADS) to classify their findings by patient disease-state [15,16,17,18,19,20,21].

## 2. Materials and Methods

### 2.1. Preparation of Training Data

**Figure 2:**
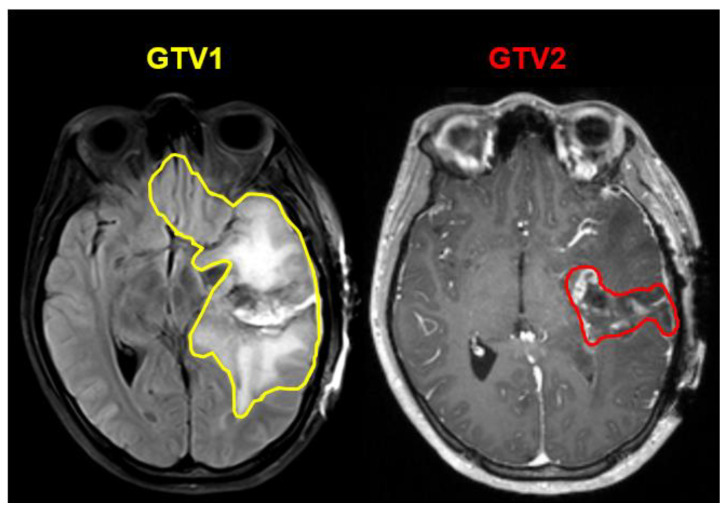
Example contour overlays from our training dataset of GTV1 (yellow contour) overlaid on T2w/FLAIR MRI and GTV2 (red contour) on CE-T1w MRI that includes cavity and blood product.

Imaging from a de-identified database of 225 GBM patients who were treated at Emory University with intensity modulated radiation therapy over the past 10 years was used to train our segmentation model. For each patient, post-operative T2w/FLAIR and CE-T1w MRIs were collected as well as their corresponding GTV1 and GTV2 contours. In Figure 2, we show an example patient in our training dataset where the extent of GTV1 includes not only the hyperintense lesion, but also cavity and blood product. In the same figure, GTV2 in CE-T1w MRI includes the cavity and residual enhancing lesion. As per standard-of-care, GTV2 typically receives a higher radiation dose of 60 Gy, while GTV1 receives a lower dose of 50–51 Gy [2,3]. Due to the range of possible margins physicians add to their GTV targets, we had a radiation oncologist resident tighten the treatment contours to only include visible lesions in both sets of images. These tightened contours were then reviewed by an independent radiation oncologist and neuroradiologist with any differences resolved by consensus until agreement was reached.

**Figure 3:**
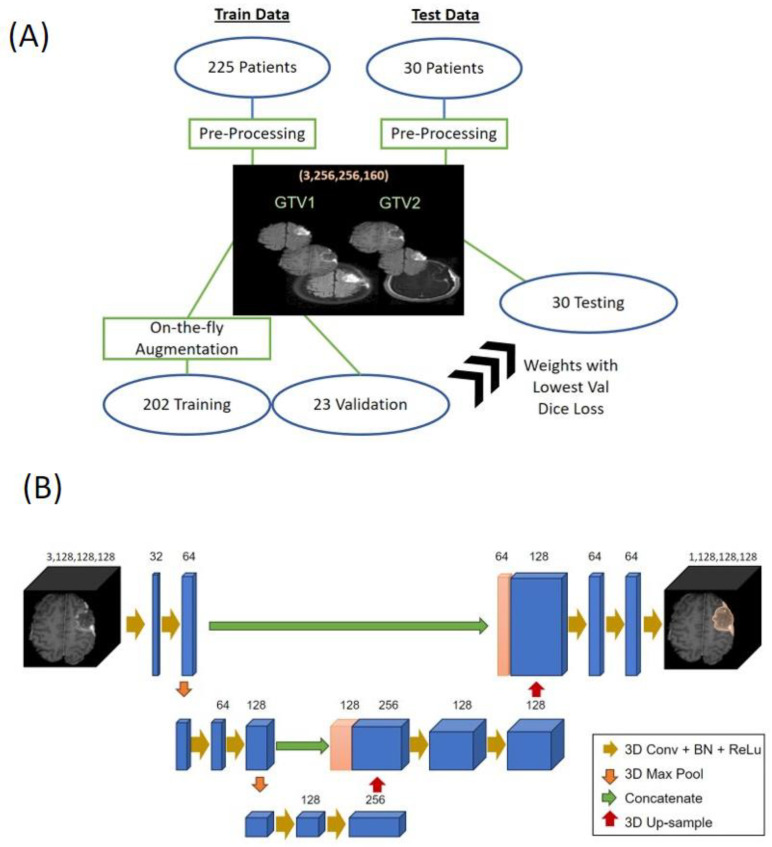
(**A**) Training pipeline for our segmentation models. During the pre-processing step, a 3-channel input was used with the first two channels using skull-stripped T2w/FLAIR and CE-T1w MRIs and the third channel including skull to assist in delineating cavity margins. The model weights that performed the best with the validation data were tested on the independent test dataset. (**B**) Network architecture for 3D Unet, which has a depth of 3, and was trained on our post-operative MRIs after pre-processing. The number of filters is listed above each block.

For image preprocessing, T2w/FLAIR MRIs were registered and interpolated to their CE-T1w counterparts. All images were skull-stripped and resampled to a (256,256,160) volume before nonzero voxels underwent zero-mean, unit-variance normalization. For GTV1 segmentation, models were fed a 3-channel input with skull-stripped T2w/FLAIR MRI as the first channel, skull-stripped CE-T1w MRI as the second channel, and with-skull T2w/FLAIR MRI as the third channel. For GTV2 segmentation, CE-T1w MRI was used for the first and third channels, while skull-stripped T2w/FLAIR was used for the middle channel (Figure 3A). A third channel with skull was included to help the model delineate the extent of resection cavities that reside close to the skull. For 2D segmentation, slices of size (256,256) were fed in batches for training, while 3D segmentation models trained on volumes of size (128,128,128). The 225-patient dataset was split into a training set of 202 patients and a validation set of 23 patients. For the training data, on-the-fly augmentation was performed with random flipping and rotation with 0.5 probability. An independent test dataset containing T2w/FLAIR and CE-T1w MRIs with corresponding GTVs for 30 post-operative GBM patients from three different sites (Emory University, Johns Hopkins University, and University of Miami) was used to evaluate segmentation performance (Figure 3A).

### 2.2. Segmentation Models and Training

We compared 2D and 3D segmentation approaches with the goal of incorporating the most robust models in clinical workflows. Three of the most popular 2D segmentation models for medical image segmentation are the standard U-net, ResUnet, and Shifted-Window (Swin)-Unet [22,23,24]. The ResUnet model has the structure of Unet but with added residual connections in the encoder and decoder blocks to prevent vanishing gradient problems, as well as atrous convolutions and pyramidal pooling to garner more information from larger receptive fields. The 2D Swin-Unet structure is similar to Unet, but replaces standard convolutional neural network (CNN)-based encoder and decoder blocks with Swin transformers, which use tokenized image patches as input and attention-based shifted-window vision transformers to garner global image features [22,25]. Finally, we compared the CNN-based 3D Unet with Swin-UNETR—both 3D segmentation models that have found great success with pre-operative brain tumor segmentation [9,10,26]. The 3D Unet is similar in structure to Unet but uses 3D filters to convolve over image volumes as inputs (Figure 3B), while Swin-UNETR uses 3D Swin transformers as encoders and standard CNNs for decoding and generating the segmentations.

For the 2D models and 3D Unet, a five-fold cross validation procedure was used to determine the optimal hyperparameters for each model [27]. We used the same hyperparameters as the authors of Swin-UNETR for brain tumor segmentation [10]. A table of key hyperparameters optimized during cross validation are in Supplemental Table S1. The Dice similarity coefficient was used to evaluate segmentation accuracy between model predictions and physician-generated segmentations and Dice loss was used during training [28]. During training, a learning rate scheduler and early stopping criteria were used to reduce learning rate based on validation accuracy to fine-tune optimization, and to stop training when validation accuracy had not improved for 10 epochs. Three-dimensional models were trained on an NVIDIA RTX 6000 with GPU memory of 48 GB, while 2D models were trained on an NVIDIA V100 with GPU memory of 32 GB. Models were then tested on the independent 30-patient test dataset to calculate test Dice scores, Hausdorff distances, and Jaccard coefficients for comparison and evaluation.

### 2.3. Application of Segmentation in Longitudinal Tracking

We incorporated our best-performing segmentation algorithms into our web application BrICS-LIT for longitudinal tracking and calculated lesion volumes for an example patient. Due to our model including the resection cavity in CE-T1w lesion segmentation, we use Otsu thresholding to cluster GTV2 segmentations into four different groups before removing the lowest intensity cluster to only include the residual enhancing lesion for longitudinal tracking purposes [29]. We then used the same volumetric percent change cutoffs used to predict RANO scores from Kickingereder et al. for BT-RADS prediction [16]. More specifically, if a T2w/FLAIR lesion changed in volume by 100% or greater or a CE-T1w lesion increased in volume by 40% or greater, then tumor had recurred and BT-RADS score of 4 was assigned. If CE-T1w lesion increased in volume by 20% or greater or T2w/FLAIR lesion volume increased by 50% or greater, then imaging was considered “worsened” and BT-RADS scores of 3 or higher were assigned. Otherwise, imaging was considered improved or stable and scores of 1 or 2 were given. Finally, if for two consecutive visits a patient’s imaging had worsened by less than the tumor recurrence cutoff, a score of 3c was assigned to the latest patient visit, suggesting they are highly likely to experience tumor recurrence. By using these cutoffs in a decision-tree-based approach, along with the lesion volumes and relevant clinical and medication information retrieved from REDCap, BrICS-LIT makes automated disease-state classifications.

## 3. Results

### 3.1. Segmentation for RT Planning

In Table 2, we compare our 2D and 3D fully automated segmentation models. When comparing the purely CNN-based 2D Unet and Resunet models to the 2D Swin-Unet model, we see a similar segmentation performance during training for both GTVs. However, the Resunet outperforms the 2D Swin-Unet model for GTV2 segmentation in the test dataset with a Dice score of 0.57. The 2D Swin-Unet model outperforms the other 2D approaches for GTV1 segmentation with a test Dice score of 0.64, which is comparable to even the 3D approaches.

While both 3D Unet and Swin-UNETR have found great success in brain tumor segmentation tasks in the past, the 3D Unet outperformed 3D Swin-UNETR for our task of segmenting radiation treatment contours and, importantly, this was including the surgical resection cavity. Interestingly, with the early stopping criteria we had in place for each training task, the Swin-UNETR model converged at a lower training Dice score of 0.64 for GTV1 and 0.65 for GTV2, whereas the 3D Unet model converged at higher GTV1 and GTV2 training Dice scores of 0.77 and 0.79, respectively. The difference in Dice scores between training and testing was at most 0.06 for the 3D models. The table also includes the Hausdorff distance and Jaccard coefficient for the test dataset. A higher Hausdorff distance indicates a greater disagreement between prediction and ground truth as it calculates the largest distance between a point in the ground truth and its closest point in the prediction, while the Jaccard coefficient generally scales with the Dice score but penalizes poor predictions more, and is therefore consistently lower in the table.

**Figure 4:**
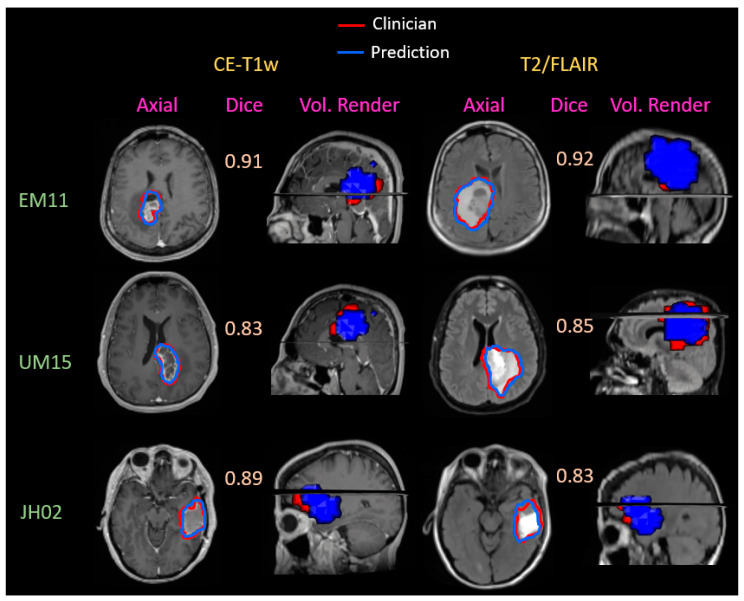
Example GTV2 (CE-T1w target) and GTV1 (T2w/FLAIR target) segmentations for patients from three different institutions with our best-performing 3D Unet model. For GTV2 and GTV1, we have included an axial view of the clinician GTVs (red) and the prediction (blue) as well as a volume-rendered view of the contour overlap in the sagittal orientation. Dice scores are included between the 2D and volume rendering for each example.

In Figure 4, we show example GTV segmentations from our best-performing model, the 3D Unet. The first two columns contain CE-T1w MRIs and the second two columns contain T2w/FLAIR MRIs for three patients from three different institutions. We show a 2D contour in axial orientation as well as a 3D volume rendering of the overlap between GTV prediction and clinician-derived contours in sagittal orientation. Our model’s GTV predictions are almost identical to those created by clinical experts, and we believe these examples illustrate the unique task that our model was trained to solve. For both GTVs, the resection cavity was included, as it typically receives a higher dose of radiation. During radiation treatment planning, clinicians can use the segmentations from CE-T1w to generate high-dose GTV2 contours and subtract the GTV2 contour from the GTV1 contour to generate a lower dose targeted towards edema and infiltrative disease.

**Figure 5:**
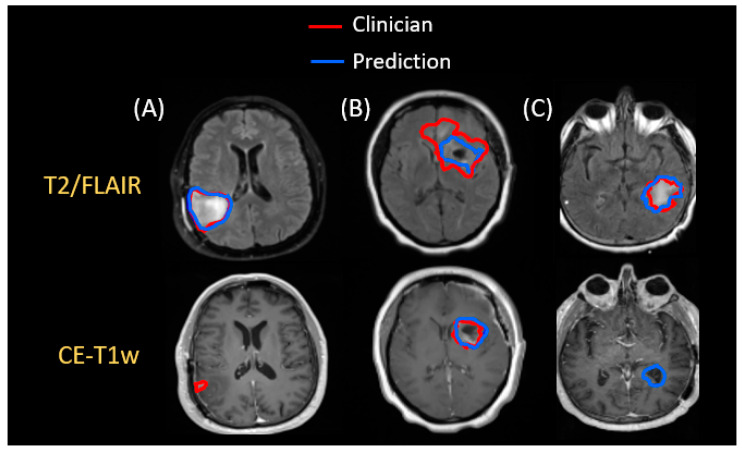
The three worst-performing cases of our 3D Unet model. In (**A**), the small CE-T1w lesion is missed, possibly being confused for blood vessel. In (**B**), the model has difficulty capturing the entire extent of hyperintensity from the 3D T2w/FLAIR acquisition, and in (**C**), the model confuses the ventricle for the shape of a cavity, especially when there are T2w/FLAIR edema in the same region.

When evaluating the performance of the 3D Unet model further, about 75% of the test patients had a Dice score greater than 0.70 for GTV1 and greater than 0.67 for GTV2. This suggests that outliers in roughly a quarter of the test dataset caused the average performance of the model to decrease. To that end, Figure 5 shows some of the poorer-performing examples. In Figure 5A, while the model appears to segment the T2w/FLAIR hyperintensity and blood product quite well, it completely misses the small enhancing lesion in CE-T1w MRI, possibly missing it due to a lack of cavity nearby or its relatively small size. Figure 5B is an example where GTV2 is segmented in CE-T1w MRI, but the model underestimates the extent of T2w/FLAIR hyperintensity. This is possibly due to the relatively low contrast between abnormal and normal tissue on some of the 3D T2w/FLAIR acquisitions. Figure 5C is a case where the model confused the ventricle for a cavity as there was hyperintense T2w/FLAIR lesion in the same area.

### 3.2. Application of Segmentation in Longitudinal Tracking

**Figure 6:**
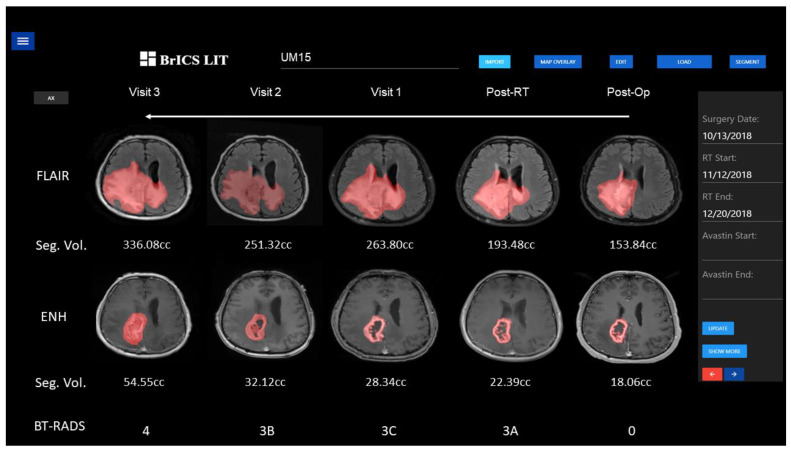
The 3D Unet segmentation model implemented into our web application BrICS-LIT, displayed for an example patient’s post-operative and post-RT follow-up MRIs. Automated lesion segmentations (red overlay) and lesion volume cutoffs assist in generating automated structured reporting scores (bottom row) for post-treatment longitudinal tracking.

With a fully automated segmentation model trained on post-operative MRIs, we sought to test our model in our web application BrICS-LIT for automated longitudinal tracking. To that end, Figure 6 shows an example patient’s pre-operative/pre-RT and post-RT follow-up MRIs. Our 3D Unet model performed segmentations of T2w/FLAIR and CE-T1w lesions, and after Otsu thresholding, the cavity was removed from the CE-T1w segmentations. Then, volumetric cutoffs that were used for RANO classification were applied to this patient’s lesion volumes. As per BT-RADS, the baseline score at post-surgery, on the far right, received a score of 0. In the next follow-up visit after the completion of RT, the CE-T1w lesion grew by almost 25% while the FLAIR lesion grew by slightly over 25%. A score of 3A was automatically given as imaging had worsened, but the visit was shortly after the completion of radiation treatment, and the increased volumes were attributed to radiation-related inflammation. In the following visit, lesion volumes increased by 37% and 27% for T2w/FLAIR and CE-T1w lesions, respectively. Due to the lesion volumes increasing for consecutive dates, a score of 3C was given, suggesting the lesion was highly likely to experience tumor recurrence. In the next visit, the T2w/FLAIR lesion volume had decreased but enhancement was similar or slightly larger. These mixed findings resulted in assigning the indeterminate BT-RADS score of 3B. On the latest visit, the CE-T1w lesion volume increased by 70%, well over the 40% cutoff, immediately causing BrICS-LIT to assign a score of 4, highly probable for tumor recurrence. This also meets the criteria for RANO progressive disease.

**Figure 7:**
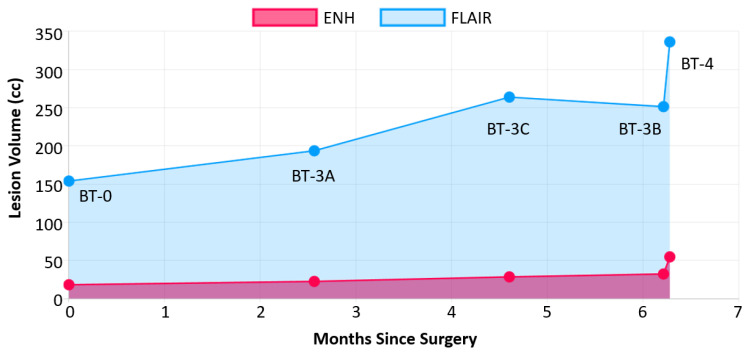
Interactive chart tracking changes in CE-T1w lesion volume (ENH), T2w/FLAIR lesion volume, and structured reporting scores for the patient shown in Figure 6. A drastic increase in lesion volume is seen 6 months after surgery.

Figure 7 shows a graph of changes in T2w/FLAIR and CE-T1w lesion over time, as well as corresponding BT-RADS scores for the same patient generated by BrICS-LIT for physician viewing. The sharp increase in both lesion volumes is apparent 6 months after surgery, signaling true tumor recurrence.

## 4. Discussion

With the advent of artificial intelligence in many domains of our daily lives, a question remains as to when it will be included in standard-of-care medical treatments. While there has been an entrepreneurial boom in integrating deep learning and machine learning to the domain of personalized medicine, there are still many challenges in including AI-based tools in clinical imaging workflows [30,31,32]. Some of these challenges include the need for physicians to have efficient image processing software that can easily pre-process clinical imaging, handle computationally heavy algorithms, and perform inference, all while requiring minimal effort by the physician [33]. Furthermore, visualization tools can help physicians understand the reasoning behind predictions and decisions that AI algorithms make [33]. To that end, we trained the first deep learning segmentation algorithm for post-operative brain tumors for the purpose of radiation treatment planning and longitudinal tracking. With reliable GTV1 and GTV2 segmentation models that can perform segmentations in less than a minute, radiation oncologists can expediently generate GTV targets and add margins depending on their preferences to generate larger CTVs and PTVs for radiation treatment. These tools could potentially be integrated into radiation planning software commonly used by radiation oncologists. By using volumetric cutoffs, we can suggest completely automated disease-state classifications for the longitudinal tracking of post-treatment brain tumor patients, which we implemented using the BT-RADS rating scale in our online BrICS-LIT platform.

In comparing our 2D and 3D segmentation approaches, it is evident that the 2D models are overtrained, as the training Dice scores were above 0.90 while the testing Dice scores ranged between 0.43–0.64. We set early stopping criteria which would cause our models to stop training when the validation accuracy failed to improve after 10 epochs. This suggests that the 2D models were still learning some generalizable information, even if the gap between training and validation accuracy was steadily increasing. The benefit of using 3D approaches is that while their training accuracy was lower than the 2D models, the testing accuracy was somewhat aligned with the training accuracy, leading us to believe the 3D approaches are more robust with test images.

Our best-performing segmentation model was the CNN-based 3D Unet, with test Dice scores of 0.72 and 0.73 for GTV1 and GTV2, respectively. While the distribution of Dice scores in the test dataset suggests that there were a few cases that severely decreased the mean Dice score, we believe this gives us room for improvement. The models struggled with small residual enhancing lesions in CE-T1w with minimal cavities, which can easily be confused for blood vessels. They also struggled with the task of differentiating cavity from ventricle, especially when there was T2w/FLAIR hyperintense lesion in the same area. Finally, the model appears to struggle with capturing the entire extent of the lesion boundary in 3D T2w/FLAIR, where the acquisitions prioritize higher spatial resolution over contrast. Although we implemented several augmentation techniques such as random intensity shifting and random k-space sampling to mimic various levels of spatial resolution, we found that augmentation did not improve our models’ ability to generalize to the test data. Future efforts will involve curating a more diverse training dataset that also includes smaller residual enhancing lesions and a higher number of 3D T2w/FLAIR acquisitions. Ensuring that models have good-quality, high-contrast 3D FLAIR acquisitions can also help with segmentation.

Many brain tumor segmentation efforts use four sets of images to help segment lesions—T1w pre-contrast MRI, CE-T1w, T2w MRI, and T2w/FLAIR. The Swin-UNETR model achieved high Dice scores with the pre-operative BRATS dataset by utilizing all four sets of images. The encoder region of the model utilizes Swin transformers, which in theory would help the model determine affinities between different regions of the image. We believe it may have underperformed with our smaller training dataset with fewer image channels due to its complexity. A model of that complexity may have overfit the dataset, and with our early stopping criteria, converged too quickly even with small learning rates compared to the 3D Unet model. We chose to train our models with only CE-T1w and T2w/FLAIR MRI in order for our models to fit within clinical workflows with minimal effort, since CE-T1w and T2w/FLAIR are the sequences most heavily relied on by radiologists, radiation oncologists, and neurosurgeons to identify tumors. When housing our models in web applications like BrICS-LIT or even attempting to integrate them in clinical software like Velocity (Varian Medical Systems), we wanted our models to have as few requirements as possible to enable easy integration. For example, our goal with BrICS-LIT is the longitudinal tracking of brain tumor patients in clinical and research settings. It is far easier for researchers and clinicians to use our models in BrICS-LIT if the models only require the imaging that the clinicians were currently viewing as inputs. We do acknowledge that extra imaging, and most importantly the T1w pre-contrast MRI, could potentially improve our models’ segmentation accuracy. To address this concern, efforts are currently underway to train generative adversarial networks (GANs) on the BraTS 2021 dataset to artificially generate T1w-pre contrast and T2w MRIs from CE-T1w and T2w/FLAIR MRIs [34,35,36]. With a complete dataset from four imaging modalities, we plan to use previous BraTS competition-winning models that are trained on pre-operative MRIs and perform transfer learning on our dataset to finetune the task of including cavities, blood product, and the entire extent of hyperintense edemas.

As demonstrated in Figure 6, we have already integrated our model in our web application (BrICS-LIT) that is actively used by clinicians and researchers. By performing automated lesion segmentation and using quantitative volumetric cutoffs, we have shown our post-operative segmentation algorithms can help us generate automated disease-state classifications for BT-RADS and RANO. While borrowing volumetric cutoffs used for RANO classification have led to interesting BT-RADS classifications, we acknowledge that both structured reporting criteria may require different volumetric cutoffs that are more suitable for each. To mitigate this issue, we have a de-identified, independent database of over 150 patients with clinician-assigned BT-RADS scores that we will treat as the gold standard to help determine new volumetric cutoffs specific to BT-RADS. Furthermore, simply using percentage changes in lesion volume has its own limitations. For example, a patient could have a residual enhancing lesion that is only 0.4 cc. In their next visit, that lesion could grow by 50% to 0.6 cc. While our automated algorithm would likely say the tumor has recurred purely based on percent change, that 0.2 cc increase in lesion volume could simply be a rounding error in our segmentation algorithm. To address this point, we are investigating the use of classification and regression tree-based machine-learning approaches to predict disease-state classifications using lesion volumes and other relevant clinical information.

## 5. Conclusions

While there have been several successful efforts in the past to segment brain tumor lesions in an automated manner from MRIs, they tend to focus on pre-operative brain MRIs, which may limit their usefulness in clinical use and in clinical trials. Here, we have trained popular medical image segmentation algorithms for post-operative, residual brain tumors for the purpose of radiation treatment planning and longitudinal tracking. The best-performing 3D Unet can segment lesions with over 70% accuracy for most of our test cases, and has already been integrated into our BrICS-LIT web application for clinician and research use.

## Figures and Tables

**Table 1:** A comparison of strengths and weaknesses between previous efforts in brain lesion segmentation and our proposed effort.

Brain Lesion Segmentation	Strengths	Weaknesses
Previous Efforts	Utilizes pre-contrast T1w and T2w MRIs. High segmentation performance for pre-surgical lesions. BraTS includes a larger training dataset from multiple institutions.	Requires more imaging for each segmentation. Cannot segment post-operative cavity.
Proposed Effort	First effort to segment post-operative lesions (including cavity) for RT planning. Model can also be used for post-RT longitudinal tracking. Longitudinal lesion volumes can be used to generate automated disease-state classifications. Ground truth contours generated and reviewed by radiation oncologists and a neuro-radiologist.	By only using CE-T1w and T2/FLAIR, models utilize less information for prediction, leading to potentially lower segmentation performance. Training dataset is smaller than BraTS, but larger than other published efforts.

**Table 2:** A comparison of 2D and 3D approaches to post-surgical GTV segmentation. The CNN-based 3D Unet outperforms the other models on our test dataset with the highest Dice scores (bolded).

Model	GTV1				GTV2			
	Train (Dice)	Test (Dice)	Test (Hausdorff)	Test (Jaccard)	Train (Dice)	Test (Dice)	Test (Hausdorff)	Test (Jaccard)
2D Unet	0.93	0.43	78.50	0.19	0.92	0.56	75.41	0.34
2D Resunet	0.93	0.58	58.50	0.36	0.91	0.57	35.57	0.35
2D Swin-Unet	0.89	0.64	60.71	0.44	0.86	0.51	35.63	0.31
3D Unet	0.77	**0.72**	12.77	0.51	0.79	**0.73**	10.75	0.58
3D Swin-UNETR	0.64	0.60	38.32	0.36	0.65	0.64	23.33	0.44

## Data Availability

The data presented in this study are available on request from the corresponding author.

## Supplementary Materials

The following supporting information can be downloaded at: https://www.mdpi.com/article/10.3390/cancers15153956/s1, Table S1: Hyperparameters for every trained model.
