# Supplementary material for: A Fully Automated Post-Surgical Brain Tumor Segmentation Model for Radiation Treatment Planning and Longitudinal Tracking
Source: Cancers (Basel). 2023 Aug 3;15(15):3956. doi: 10.3390/cancers15153956 (PMC10417353; doi:10.3390/cancers15153956)
Supplement: Supplementary file 1 [file cancers-15-03956-s001.zip › cancers-2505661-supplementary.pdf]

Supplemental Table S1. Hyperparameters for every trained model.

Model	Learning Rate	Dropout Rate	Batch Size
2D Unet	0.0001	0.2	32
2D Resunet	0.0001	0.3	16
2D Swin-Unet	0.0001	0.1	32
3D Unet	0.001	0.1	2
3D Swin-UNETR	0.0008	0.1	2
